# Estimating classification accuracy in positive-unlabeled learning: characterization and correction strategies

**Published:** 2019

**Authors:** Rashika Ramola, Shantanu Jain, Predrag Radivojac

**Affiliations:** Northeastern University, Boston, Massachusetts, U.S.A.

**Keywords:** Positive-unlabeled learning, AlphaMax, Matthews correlation, accuracy estimation

## Abstract

Accurately estimating performance accuracy of machine learning classifiers is of fundamental importance in biomedical research with potentially societal consequences upon the deployment of best-performing tools in everyday life. Although classification has been extensively studied over the past decades, there remain understudied problems when the training data violate the main statistical assumptions relied upon for accurate learning and model characterization. This particularly holds true in the open world setting where observations of a phenomenon generally guarantee its presence but the absence of such evidence cannot be interpreted as the evidence of its absence. Learning from such data is often referred to as positive-unlabeled learning, a form of semi-supervised learning where all labeled data belong to one (say, positive) class. To improve the best practices in the field, we here study the quality of estimated performance in positive-unlabeled learning in the biomedical domain. We provide evidence that such estimates can be wildly inaccurate, depending on the fraction of positive examples in the unlabeled data and the fraction of negative examples mislabeled as positives in the labeled data. We then present correction methods for four such measures and demonstrate that the knowledge or accurate estimates of class priors in the unlabeled data and noise in the labeled data are sufficient for the recovery of true classification performance. We provide theoretical support as well as empirical evidence for the efficacy of the new performance estimation methods.

## 1. Introduction

Machine learning-based prediction has become the cornerstone of modern computational biology and biomedical data science. Numerous approaches have been developed and applied in these fields, including those related to the function of biological macromolecules,^1,2^ the effect of genomic variation,^3^ precision medicine,^4,5^ or computer-aided clinical decision making.^6^ A significant part of this research considers binary classification where the learning algorithms have been extensively studied and characterized, both theoretically and empirically.^7^ The objective in binary classification is to train (learn) a model (function) that can distinguish one type of objects from another; e.g., predicting the effect of single nucleotide variants as pathogenic or benign.^3^ However, these algorithms have a broader value because multi-class, multi-label and even structured-output learning are often framed as extensions of binary classification, sometimes in a straightforward manner.^8^

In addition to learning, binary classification has also been extensively studied with respect to the performance evaluation of predictive models.^7^ Typically, the prediction algorithm outputs a real-valued score for a given input example, after which a thresholding function is applied to map the prediction score into one of the elements of the output space (e.g., pathogenic vs. benign). In some cases, one first chooses the decision threshold and then computes the performance measures for the model on the binarized predictions. In others, calculating the performance measures entails some form of aggregating over all decision thresholds. The first category of evaluation metrics includes classification accuracy, or the probability that a randomly selected, previously unseen, example from the population will be correctly classified. Other, more specialized measures, include the true positive rate (sensitivity, recall), true negative rate (specificity, 1 − false positive rate) or precision (positive predictive value, 1 − false discovery rate).^7^ These measures may also be combined to compute derived quantities such as the balanced sample accuracy, F-measure^7^ or Matthews correlation coefficient.^9^ The second group of metrics include two-dimensional plots such as the Receiver Operating Characteristic (ROC) curve and the precision-recall curve that visualize the trade-offs between various quantities as a function of the decision threshold. These curves can be further summarized into a single quantity by computing the area under the curve. Alternatively, metrics such as F-measure can be computed for each decision threshold to report the maximum value over all thresholds; e.g., *F*_max_.^10^ This allows each algorithm to select its own decision threshold and also comparisons between algorithms that binarize their outputs with those that do not. It is worth mentioning that cost-sensitive learning and evaluation,^11,12^ as well as information-theoretic approaches^13,14^ can also be considered in certain classification scenarios; however, these evaluation strategies are beyond the scope of this work.

Although binary classification has been extensively studied and is well understood,^7^ there remain problems related to the open world setting that require attention. Open world refers to the framework in knowledge representation and artificial intelligence in which the observation of a phenomenon generally establishes its presence; however, the lack of the observation cannot be interpreted as the evidence of absence of the phenomenon. One such example is protein function assignment,^15^ where an experimental assay can definitively establish, say, that a particular protein is an enzyme. High-throughput experiments can similarly establish the presence of the phenomenon, albeit with some error as in generating protein-protein interaction networks using yeast two-hybrid systems.^16^ However, no protein has ever been experimentally assayed for all functions and, additionally, an unsuccessful experiment does not necessarily establish the lack of particular activity. This is because an absence of required molecular partners, an inadequate set of experimental conditions (e.g., pH, temperature^17^), or a human error can combine to result in a failed experiment.^b^ When presented with such data, one is *de facto* given a set of positive examples (e.g., enzymes) and a set of unlabeled examples (e.g., a sample of all proteins) and the learning setting is referred to as positive-unlabeled learning.^18^ Although the unlabeled set contains an unknown fraction of positive examples, the standard practice ignores this fact and considers all unlabeled examples to be negative. One then trains a prediction model (interestingly, this approach is optimal for a wide range of loss functions referred to as composite loss functions^19^) and estimates its performance, after which the predictor is deployed with a particular estimated quality. In other words, machine learning models in the positive-unlabeled setting are trained/evaluated on positive vs. unlabeled data, whereas the ideal predictor, certainly one expected by the downstream user, would be trained/evaluated on positive vs. negative data. Following Elkan and Noto,^20^ we will refer to the predictors trained on positive vs. negative data as traditional classifiers and models trained on positive vs. unlabeled data as non-traditional classifiers. Similarly, we will refer to the two different types of evaluation as traditional and non-traditional evaluation.

The primary objective of this work is to study non-traditional classifiers and the adverse effects of non-traditional performance evaluation when the intent is to carry out a traditional evaluation. We show that the traditional performance of these classifiers can be recovered with the knowledge or an accurate estimate of class priors (i.e., the fractions of the positive and negative examples in a representative unlabeled set) and the labeling noise (i.e., the fraction of negative examples in the labeled data set that have been mistakenly labeled as positive). We conduct extensive and systematic experiments to evaluate the proposed methods and draw conclusions pertaining to the best practices of performance evaluation in the field.

## 2. Methods

### 2.1. Performance measures: definitions and estimation

In this section, we give definitions of several widely used performance measures and their standard estimation formulas. To this end, we first describe the probabilistic framework used in the definitions. Consider a binary classification problem of mapping an input x∈X to its class label y∈Y = {0,1}. Assume that *x* and *y* come from an underlying, fixed but unknown joint distribution *h*(*x,y*) over X×Y.^c^ Let *h*(*x*) denote its marginal density over *x*. It follows that *h*(*x*) can be expressed as a two-component mixture:

(1) $$h(x)\;=\;\pi h_{1}(x)\;+\;(1\;-\;\pi )h_{0}(x),$$

for all x∈X, where *h*_1_ and *h*_0_ represent the distributions of the positive and negative examples (inputs), respectively, and *π* ∈ (0,1) is the proportion of positive examples in *h*, also referred to as the class prior for the positive class.

Next, we give definitions of the three most fundamental performance measures: (1) true positive rate (*γ*), the probability that a positive example is correctly classified, (2) false positive rate (*η*), the probability that a negative example is incorrectly classified as positive, and (3) precision (*ρ*), the probability that a positive prediction is correct. Mathematically, given a binary classifier $\hat{y}:X\to Y$, they are defined as

(2) $$\gamma \;=\;E_{h_{1}}[\hat{y}(x)],\;\eta \;=\;E_{h_{0}}[\hat{y}(x)],\;\rho \;=\;\frac{\pi E_{h_{1}}[\hat{y}(x)]}{E_{h}[\hat{y}(x)]}\;=\;\frac{\pi \gamma }{\theta }$$

where $E_{h}$ denotes expectations w.r.t. *h* and $\theta \;=\;E_{h}[\hat{y}(x)]$ is the probability of a positive prediction. A classifier with a high *γ* and *ρ*, but low *η* is desirable. However, these measures are at odds with each other; i.e., typically, increasing a classifier’s *γ* leads to a smaller *ρ* and a larger *η*. A classifier that always predicts either 0 or 1 can optimize them individually at the expense of others. Consequently, they are often used together to gauge a classifier’s performance; for example, in an ROC curve analysis. Moreover, other performance measures combine them explicitly or implicitly in their formulation. Though *θ* itself is not widely used as a measure of classifier performance, it also appears in the expression of several important measures (a classifier for which *θ > π* is sometimes said to “overpredict”). A particularly useful expression of *θ* in terms of *γ,η* and *π* is derived as follows.

(3) $$\theta \;=\;E_{h}[\hat{y}(x)]\;=\;\pi E_{h_{1}}[\hat{y}(x)]\;+\;(1\;-\;\pi )E_{h_{0}}[\hat{y}(x)]\;=\;\pi \gamma \;+\;(1\;-\;\pi )\eta$$

In this paper, we focus on four performance measures that are widely used in biomedical research: (1) Accuracy (acc), the probability that a random example is correctly classified (2) Balanced accuracy (bacc), the average accuracy on the positive and negative examples, weighed equally, (3) F-measure (*F*), the harmonic mean of *γ* and *ρ*,^d^ and (4) Matthews correlation coefficient (mcc), the correlation between the true and predicted class. Mathematically, they are defined as follows:

(4) acc = πγ + (1 − π)(1 − η)

(5) $$\text{bacc }=\;\frac{1\;+\;\gamma \;-\;\eta }{2}$$

(6) $$F\;=\;\frac{1}{\frac{1}{2}\cdot \frac{1}{\gamma }+\frac{1}{2}\cdot \frac{1}{\rho }}\;=\;\frac{2\pi \gamma }{\pi \;+\;\theta }$$

(7) $$\text{mcc }=\;\frac{E_{h}[y\cdot \hat{y}(x)]\;-\;E_{h}[y]\;\cdot \;E_{h}[\hat{y}(x)]}{\sqrt{V_{h}[y]\;\cdot \;V_{h}[\hat{y}(x)]}}$$

where $V_{h}$ in Eq. (7) denotes the variance operator w.r.t. distribution *h*(*x*). Notice that, since *y* ~ Bernoulli(*π*) under *h*, $E_{h}[y]\;=\;\pi$ and $V_{h}[y]\;=\;\pi (1\;-\;\pi )$; similarly, $V_{h}[\hat{y}(x)]\;=\;\theta (1\;-\;\theta )$. Further, using the law of iterated expectations, $E_{h}[y\cdot \hat{y}(x)]\;=\;\pi E_{h_{1}}[\hat{y}(x)]\;=\;\pi \gamma$. Thus,

(8) $$\text{mcc }=\sqrt{\frac{\pi }{\left(1\;-\;\pi \right)}}\frac{\gamma \;-\;\theta }{\sqrt{\theta (1\;-\;\theta )}}=\sqrt{\frac{\pi (1\;-\;\pi )}{\theta (1\;-\;\theta )}}\cdot (\gamma \;-\;\eta )$$

Using the estimates of *γ*, *η*, *π* and *θ* from Table 1, we give the standard formulas for acc, bacc, *F* and mcc estimation, in terms of the classifier’s confusion matrix entries. For example, simple algebraic operations on Eq. (8) give

$$\hat{\text{mcc}}\;=\;\frac{\hat{\pi }(1\;-\;\hat{\pi })(\hat{\gamma }\cdot (1\;-\;\hat{\eta })\;-\;\hat{\eta }\cdot (1\;-\;\hat{\gamma }))}{\sqrt{\hat{\theta }\hat{\pi }(1\;-\;\hat{\pi })(1\;-\;\hat{\theta })}}\;=\;\frac{t\text{p}\cdot tn\;-\text{ fp}\cdot \text{fn}}{\sqrt{\left(\text{tp }+\text{ fp})(\text{tp }+\text{ fn})(\text{tn }+\text{ fn}\right)}}$$

Similarly, the standard estimation formulas for acc, bacc and *F* can be easily derived as:

$$\hat{\text{acc}}\;=\;\frac{\text{tp }+\text{ tn}}{\text{tp }+\text{ fn }+\text{ tn }+\text{ fp}},\;\hat{\text{bacc}}\;=\;\frac{1}{2}\frac{\text{tp}}{\text{tp}+\text{fn}}\;+\;\frac{1}{2}\frac{\text{tn}}{\text{tn}+\text{fp}},\;\hat{F}\;=\;\frac{2\text{tp}}{2\text{tp }+\text{ fn }+\text{ fp}}.$$

### 2.2. Positive-unlabeled setting

Let **D** represent a set of examples drawn from *h*(*x*); at this stage, the class of an *x* in **D** is unknown. Consider a labeling procedure that selects some examples from **D** for labeling. As is the case in many domains, the procedure tests only for the class of interest, the positive class. The procedure is successful when it deems the example as positive with high confidence. The successfully labeled examples are collected in a labeled set **L**, whereas the rejected examples along with the examples not selected for labeling, in the first place, are collected in an unlabeled set **U**. In spite of being labeled as positive, some examples in **L** might, in fact, be negative, due to the errors in the labeling procedure.

The typical, positive-unlabeled assumption made about the labeler is that the examples from **D** are selected independently of *x*, given *y* and further, that the same assumptions apply to the success of labeling.^20,21^ The assumptions ensure that the distributions of positives and negatives remain unchanged in **L** and **U** and only the class proportions are affected. Let *f*(*x,y*) and *g*(*x,y*) denote the underlying joint distribution of **U** and **L**, respectively. Note that *y* still denotes the true unobserved class and not class assigned by the labeler. For *f*(*x*) and *g*(*x*) denoting the marginals over *x*,

(9) $$f(x)\;=\;\alpha h_{1}(x)\;+\;(1\;-\;\alpha )h_{0}(x),\;g(x)\;=\;\beta h_{1}(x)\;+\;(1\;-\;\beta )h_{0}(x),$$

for all x∈X, where *α* and *β* denote the proportion of positives in the unlabeled and labeled set, respectively. By design, **L** has a higher concentration of positives than **D**; i.e., *β* ∈ (*π*,1]. Similarly, **U** has a lower concentration of positives than **D**; i.e., *α* ∈ [0*,π*). When *β* = 1 we say that the labeled data is clean. When *β <* 1, the labeled data contains a fraction (1 − *β*) of negatives that are mislabeled. We will refer to the latter scenario as the noisy positive setting and 1 − *β* as the noise proportion.

The relationship between *h*, *f* and *g* is further constrained, since **D** is partitioned by **L** and **U**. Precisely,

(10) $$h(x)\;=\;cg(x)\;+\;(1\;-\;c)f(x)\;=\;(c\beta \;+\;(1\;-\;c)\alpha )h_{1}(x)\;+\;(1\;-\;c\beta \;-\;(1\;-\;c)\alpha )h_{0}(x),$$

for all x∈X, where $c\;=\;\frac{\left|L\right|}{\left|L|+|U\right|}$. Thus,

(11) π = cβ + (1 − c)α.

To distinguish *h* from *f* and *g*, we refer to *h* as the true or the target distribution. We are primarily interested in a classifier’s performance on the true distribution, which is reflected in our goal to obtain unbiased estimates of the performance measures w.r.t. the true distribution.

### 2.3. Performance measure correction

The absence of negative examples in positive-unlabeled learning is tackled by treating the unlabeled set as a surrogate for negatives. This is referred to as the non-traditional approach.^20^ A non-traditional classifier trained on such data learns to discriminate the labeled-as-positive set from the unlabeled set. Surprisingly, an optimal non-traditional classifier has been shown to perform optimally in the traditional sense; i.e., as a discriminator between the positive and negative examples.^21^ However, measuring a classifier’s performance non-traditionally does not reflect its performance in the traditional sense. Ref. 22 demonstrated the bias in the nontraditionally estimated *γ*, *η* and *ρ* and its implications towards the ROC and precision-recall analysis. They also provided techniques for bias correction using estimates of the class prior and the noise proportion.^22^ We take a similar approach in this work and show that the standard estimators of acc, bacc*, F* and mcc, when used in a non-traditional framework, are biased. Then we give formulas to correct the bias by estimating the class prior and the noise proportion. To formalize the notion of a non-traditional labeled set, we introduce the pseudo class $\tilde{y}$, which is 1 for every example in **L** and 0 for those in **U**. The non-traditional labeled set $L^{\text{pu}}$ contains all examples from **L** and **U** along with their pseudo class labels. The standard approach (see Table 1) for estimating *γ*, *η*, *π* and *θ* presupposes that the examples in the labeled set are drawn randomly from *h*(*x,y*) and more importantly, that tp, fn, tn and fp are counted w.r.t. the true class. However, when working with $L^{\text{pu}}$, the counts are based on the pseudo class, which affects the quality of the standard estimates.

In particular, $\hat{\gamma }$ and $\hat{\eta }$ give biased estimates of *γ* and *η*, respectively. Instead, they give unbiased estimates of $\gamma^{\text{pu}}\;=\;E_{g}[\hat{y}(x)]$ and $\eta^{\text{pu}}\;=\;E_{f}[\hat{y}(x)]$; this is because *g* and *f* correspond to the distributions of the pseudo positives and the pseudo negatives, respectively. Moreover, $\hat{\pi }$ represents the proportion of the pseudo positives *c*, instead of *π*; that is, $\hat{\pi }\;=\;c$. However, $\hat{\theta }$ is still an unbiased estimator of *θ*, since *θ* only depends on the marginal distribution of *x* in $L^{\text{pu}}$, which is the same as *h*(*x*) as per Eq. (10). To summarize, we have

$$\hat{\gamma }\overset{\;}{\underset{\to }{\text{estimates}}}\;\gamma^{\text{pu}}\neq \gamma ,\;\hat{\eta }\underset{\to }{\text{ estimates}}\;\eta^{\text{pu}}\neq \eta ,\;\hat{\pi }=c\neq \pi ,\;\hat{\theta }\;\underset{\to }{\text{estimates}}\;\theta .$$

The bias in $\hat{\gamma }$, $\hat{\eta }$ and $\hat{\pi }$ is also reflected in the standard estimates of acc, bacc, *F* and mcc. They give unbiased estimates of the following quantities instead.

$${\text{acc}}^{\text{Pu}}\;=\;c\gamma^{\text{pu}}\;+\;(1\;-\;c)\left(1\;-\;\eta^{\text{pu}}\right)$$

$${\text{bacc}}^{\text{pu}}\;=\;\frac{1\;+\;\gamma^{\text{pu}}\;-\;\eta^{\text{pu}}}{2}$$

$$F^{\text{pu}}\;=\;\frac{2c\gamma^{\text{pu}}}{c\;+\;\theta }$$

$${\text{mcc}}^{\text{pu}}\;=\;\sqrt{\frac{c(1-c)}{\theta (1-\theta )}}\cdot \left(\gamma^{\text{pu}}\;-\;\eta^{\text{pu}}\right)$$

Next, we give the relationship between *γ*, *η*, *γ*^pu^ and *η*^pu^ which are then used for bias correction.

$$\gamma \;=\;\frac{(1\;-\;\alpha )\gamma^{\text{pu}}\;-\;(1\;-\;\beta )\eta^{\text{pu}}}{\beta \;-\;\alpha }$$

obtained by solving

$$\gamma^{\text{pu}}\;=\;E_{g}[\hat{y}(x)]\;=\;\beta \gamma \;+\;(1\;-\;\beta )\eta$$

$$\eta \;=\;\frac{\beta \eta^{\text{pu}}\;-\;\alpha \gamma^{\text{pu}}}{\beta \;-\;\alpha }$$

$$\eta^{\text{pu}}\;=\;E_{f}[\hat{y}(x)]\;=\;\alpha \gamma \;+\;(1\;-\;\alpha )\eta$$

We derive the bias-corrected estimates of acc, bacc*, F* and mcc by correcting for *γ,η* and *π*:

(12) $${\hat{\text{acc}}}_{cr}\;=\;{\hat{\pi }}_{cr}{\hat{\gamma }}_{cr}\;+\;\left(1\;-\;{\hat{\pi }}_{cr}\right)\left(1\;-\;{\hat{\eta }}_{\text{cr}}\right)$$

(13) $${\hat{\text{bacc}}}_{cr}\;=\;\frac{1\;+\;{\hat{\gamma }}_{cr}\;-\;{\hat{\eta }}_{cr}}{2}$$

(14) $${\hat{F}}_{cr}\;=\;\frac{2{\hat{\pi }}_{cr}{\hat{\gamma }}_{cr}}{{\hat{\pi }}_{cr}\;+\;\hat{\theta }}$$

(15) $${\hat{mcc}}_{cr}\;=\;\sqrt{\frac{{\hat{\pi }}_{cr}\left(1\;-\;{\hat{\pi }}_{cr}\right)}{\hat{\theta }(1\;-\;\hat{\theta })}}\left({\hat{\gamma }}_{cr}\;-\;{\hat{\eta }}_{cr}\right)$$

Where ${\hat{\gamma }}_{cr}$, ${\hat{\eta }}_{cr}$ and ${\hat{\pi }}_{cr}$ are estimated using estimates of *α* and *β* as follows:

$${\hat{\gamma }}_{cr}\;=\;{\left(\hat{\beta }\;-\;\hat{\alpha }\right)}^{-1}((1\;-\;\hat{\alpha })\hat{\gamma }\;-\;(1\;-\;\hat{\beta })\hat{\eta }),\;{\hat{\eta }}_{cr}\;=\;{\left(\hat{\beta }\;-\;\hat{\alpha }\right)}^{-1}(\hat{\beta }\hat{\eta }\;-\;\hat{\alpha }\hat{\gamma }),\;{\hat{\pi }}_{cr}\;=\;c\hat{\beta }\;+\;(1\;-\;c)\hat{\alpha }.$$

Theorem 2.1 shows that unbiased bacc and mcc estimates can also be directly recovered from bacc^pu^ and mcc^pu^ estimates, requiring only estimation of classifier-independent quantities *π,α* and *β* (the class proportions in **D**, **U** and **L**); i.e., *γ* and *η* do not need to be corrected as an intermediate step. Furthermore, the relationship between bacc (mcc) and its positive-unlabeled counterpart is monotonic, which is a desirable property when constructing a classifier by thresholding a score function. It ensures that the threshold obtained with the positive-unlabeled data by optimizing the non-traditional measure also maximizes the traditional measure. The inequalities derived in the theorem demonstrate that the nontraditionally evaluated bacc and mcc underestimate the traditional performance, provided the non-traditional classifier performs better than random.

**Theorem 2.1.** *The following equations hold true.*

$$\text{bacc }=\;\frac{2{\text{bacc}}^{\text{pu}}\;-\;1}{2(\beta \;-\;\alpha )}\;+\;\frac{1}{2},$$

*and*

$$mcc\;=\;\frac{1}{\beta \;-\;\alpha }\sqrt{\frac{\pi (1-\;\pi )}{c(1\;-\;c)}}\cdot {\text{mcc}}^{\text{pu}}$$

*Moreover,*

$$sign(\text{mcc})\left(\text{mcc }-\;{\text{mcc}}^{\text{pu}}\right)\geq 0,$$

and

bacc − baccpu ≥ 0, when bacc pu ≥ 12.

**Proof.** The proof of the two equalities follow by observing *γ*^pu^ −*η*^pu^ = (*β*−*α*)(*γ*−*η*) and using it in the expressions of bacc^pu^ and mcc^pu^, thereby obtaining a conversion to bacc and mcc (Eqs. (5) and (8)). Now, $\text{mcc }-\;{\text{mcc}}^{\text{pu}}\;=\;{\text{mcc}}^{\text{pu}}\left(\frac{1}{\beta -\alpha }\sqrt{\frac{\pi (1-\pi )}{c(1-c)}}\;-\;1\right)$. The mcc inequality follows since $\sqrt{\frac{\pi }{c(\beta \;-\;\alpha )}}\cdot \sqrt{\frac{1\;-\;\pi }{\left(1\;-\;c)(\beta \;-\;\alpha \right)}}\geq 1$ because *π*−*c*(*β*−*α*) = *α* ≥ 0 and 1−*π*−(1−*c*)(*β*−*α*) = 1−*β* ≥ 0. The bacc inequality follows since *β* − *α* ≥ 0 and consequently, $2\text{bacc }-\;2{\text{bacc}}^{\text{Pu}}\;=\;\frac{2{\text{bacc}}^{\text{pu}}\;-\;1}{\beta \;-\;\alpha }\;-\;\left(2{\text{bacc}}^{\text{pu}}\;-\;1\right)\geq 0$, provided bacc^pu^ ≥ 1*/*_2_. ◻

## 3. Experiments and Results

### 3.1. A case study

We first demonstrate the problem with non-traditional evaluation in a situation where the positive and negative conditional distributions, *h*_1_ and *h*_0_, are univariate Gaussians with $E_{h_{1}}[x]>E_{h_{0}}[x]$ and $V_{h_{1}}[x]\;=\;V_{h_{0}}[x]$. Knowing the underlying distributions allows us to make exact computations of performance measures, instead of estimating them from data. As per Section 2, let *h*(*x*) = *πh*_1_(*x*)+(1−*π*)*h*_0_(*x*), *f*(*x*) = *αh*_1_(*x*)+(1−*α*)*h*_0_(*x*) and *g*(*x*) = *βh*_1_(*x*)+(1−*β*)*h*_0_(*x*) be the true, labeled and unlabeled data distributions, respectively. Values of *α*, *β* and *c* will be fixed, from which *π* = *cβ*+(1−*c*)*α* will be computed. We will consider a simple linear classifier $\hat{y}(x)\;=\;1(x\geq \tau )$, where 1(·) is the indicator function and τ∈ℝ is the decision threshold. This thresholding function predicts a 0 for inputs below *τ*; otherwise, it predicts a 1.

In the traditional setting, the true positive rate (*γ*) and false positive rate (*η*) can be straightforwardly computed as $\gamma \;=\;1\;-cdf_{h_{1}}(\tau )$ and $\eta \;=\;1\;-\;cdf_{h_{0}}(\tau )$, where cdf_*f*_ is the cumulative distribution function corresponding to the density *f*. On the other hand, when evaluated in the non-traditional setting, these quantities can be expressed as *γ*^pu^ = 1 − cdf_*g*_(*τ*) and *η*^pu^ = 1 − cdf_*f*_(*τ*). The probability of positive prediction *θ* is computed using Eq. (3). Of course, *g* = *h*_1_ when *β* = 1 and *f* = *h*_0_ when *α* = 0, but this case corresponds to the standard supervised learning problem and is not of interest.

**Fig. 1:**
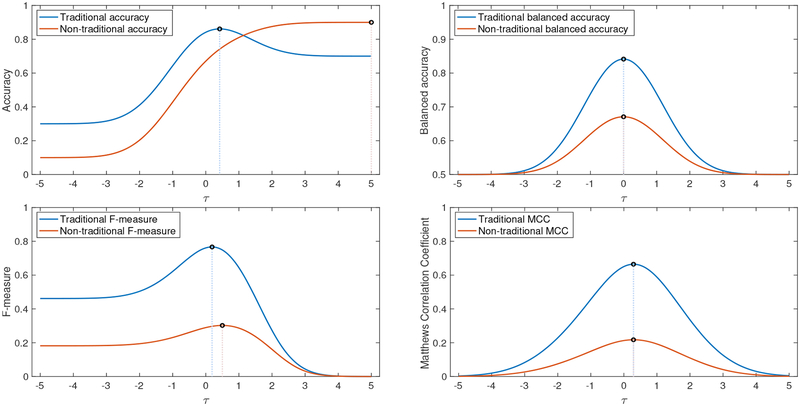
Traditional vs. non-traditional performance accuracy as a function of decision threshold *τ*. The circles and vertical lines in all four panels indicate the threshold values and the corresponding best performances in both traditional and non-traditional setting. (Upper left) Classification accuracy: top traditional performance acc_max_ = 0.86 is reached at the threshold value *τ* = 0.42, whereas the top non-traditional performance ${\text{acc}}_{\text{max}}^{\text{pu}}\;=\;0.90$ is reached at *τ* = 5; (Upper right) Balanced accuracy: top traditional performance bacc_max_ = 0.84 and non-traditional performance ${\text{bacc}}_{\text{max}}^{\text{pu}}\;=\;0.67$ are both reached at *τ* = 0; (Lower left) F-measure: top traditional performance *F*_max_ = 0.77 is reached at *τ* = 0.19, whereas the top non-traditional performance $F_{\text{max}}^{\text{pu}}\;=\;0.30$ is reached at *τ* = 0.50; (Lower right) Matthews Correlation Coefficient: top traditional performance mcc_max_ = 0.66 and non-traditional performance ${\text{mcc}}_{\text{max}}^{\text{pu}}\;=\;0.22$ are both reached at *τ* = 0.29.

Let us now be concrete and consider that $h_{0}\;=\;N(-1,1)$, $h_{1}\;=\;N(1,1)$, α = 12, β = 34 and c = 110; thus, π = 310. In Figure 1, we plot the values of the accuracy, balanced accuracy, F-measure and Matthews correlation coefficient in the traditional and non-traditional setting for each value of *τ* ∈ (−5,5), where acc, acc^pu^, bacc, bacc^pu^, *F*, *F*^pu^, mcc and mcc^pu^ are calculated from *γ*, *η*, *θ*, *h*, *f*, *g*, and *c*, as shown in Section 2. As a reminder, *c* represents the proportion of labeled examples in the training set consisting of all labeled and unlabeled examples; however, a data set is not generated here. It is important to point out the large differences between all traditional and non-traditional estimates, which provide evidence that the non-traditional estimates can be far from accurate, as in this example. As proved in Section 2, the maximum values for ${\text{bacc}}_{\text{max}}\text{ vs}{\text{. bacc}}_{\text{max}}^{\text{pu}}$ and ${\text{mcc}}_{\text{max}}\text{ vs}{\text{. mcc}}_{\text{max}}^{\text{pu}}$ are observed at the same score thresholds *τ*, respectively. This is desirable as one can establish the best decision threshold using positive-unlabeled data and secure the best predictor performance even without the precise knowledge of what that performance is. On the other hand, ${\text{acc}}_{\text{max}}\text{ vs}{\text{. acc}}_{\text{max}}^{\text{pu}}$ as well as $F_{\text{max}}\text{ vs. }F_{\text{max}}^{\text{pu}}$ do not occur at the same decision thresholds, which presents a problem for method benchmarking. The F-measure is further interesting as a simple predictor (*τ* = −5) that gives positive predictions on (almost) all inputs can achieve a high-scoring *F*, which may be misinterpreted in practice as good performance. Similarly, in terms of accuracy, an inability to “beat” a trivial classifier (the one always predicting the majority class) might be incorrectly interpreted as inability to develop a good classifier.

### 3.2. Data sets

The empirical evaluation was carried out on 14 data sets from the UCI Machine Learning repository. The selected data sets span various biomedical problems, such as recognizing splice-junction boundaries from the DNA sequence,^23^ predicting the physical activity of an individual based on their smartphone^24^ or sensor^25^ data, and predicting hospital re-admissions by using a patient’s demographics, medical diagnoses and lab test results.^26^ Where necessary, the data sets were converted to binary classification problems by considering one of the classes as positive and the other(s) as negative or by converting regression problems to classification by introducing appropriate thresholds on the target variable. The following data sets were used: Covertype, Activity recognition with healthy older people using a batteryless wearable sensor (two experiments), Epileptic Seizure Recognition, Smartphone-Based Recognition of Human Activities and Postural Transitions, Mushroom, Thyroid Disease, Anuran Calls, Wilt, Abalone, HIV-1 protease cleavage, Splice-junction Gene Sequences, Parkinsons Telemonitoring, and Physicochemical Properties of Protein Tertiary Structure.

### 3.3. Experimental protocols

The experiments were designed to simulate the construction of non-traditional classifiers in the positive-unlabeled setting and assess the quality of performance estimation both in the non-traditional and traditional mode. Labeled and unlabeled data sets, with *n*_*l*_ and *n*_*u*_ examples, respectively, were first created by sampling an appropriate number of positive/negative examples as follows. After fixing the value of *β* from {1, 0.9, 0.8, 0.7}, *β* ·*n*_*l*_ points were sampled from the positive set and (1 − *β*) · *n*_*l*_ from the negative set to make the labeled data set. This process determined the true value of *α* as the ratio of the remaining positive points and the remaining negative points from the original data set. Unlabeled data set was then formed by selecting *α* · *n*_*u*_ points from the remaining positive points and (1 − *α*) · *n*_*u*_ points from the remaining negative points. The number of unlabeled examples *n*_*u*_ was set to 10,000 in all data sets with sufficient size. Otherwise, it was set to 5000, 2000 or 1000. The size of the labeled data set *n*_*l*_ was picked so as to fix the ratio of labeled vs. unlabeled data to 1:10. That is, if *n*_*u*_ = 1000, *n*_*l*_ would be set to 100. This ratio mimics a typical situation in which one is presented with larger unlabeled data compared to the labeled data. A non-linear classification model was trained on each non-traditional data set. Its performance was evaluated in both non-traditional and traditional setting. This experiment was repeated 50 times for different random selections of labeled and unlabeled data sets, each of which was considered for four different values of *β*.

One-hundred bagged two-layer neural networks, each with 7 hidden neurons, were used as a non-traditional classifier in all experiments. The networks were trained using the RPROP algorithm^27^ with a validation (25% of the training set) stop or at most 5,000 epochs. Out-of-bag performance evaluation was carried out in all experiments. At the end of each run, we calculated four performance measures: the maximum classification accuracy (acc_max_), the maximum balanced accuracy (bacc_max_), the maximum F-measure (*F*_max_) and the maximum MCC (mcc_max_), in four different scenarios: (1) the non-traditional (PU) estimates, where the labeled data was considered to be positive and unlabeled data negative; (2) the traditional (true) performance estimates, where the actual class labels instead of the PU labels were used; (3) the recovery setting proposed in Section 2 with actual (*α, β*) values; and (4) the recovery setting proposed in Section 2 with estimated (*α, β*) values, referred to as $\left(\hat{\alpha },\hat{\beta }\right)$. The non-traditional estimates provide the performance that a practitioner would report by ignoring noise and assuming that the unlabeled set was negative. The traditional performance estimates represent the estimated true performance of these models that a practitioner would not be aware of. The third and fourth scenario represent the traditional estimates after the correction. They were designed to explore the effects of incorrectly estimating (*α, β*), instead of knowing their true values. The AlphaMax algorithm^21,28^ was used to obtain $\left(\hat{\alpha },\hat{\beta }\right)$.

### 3.4. Results

**Fig. 2:**
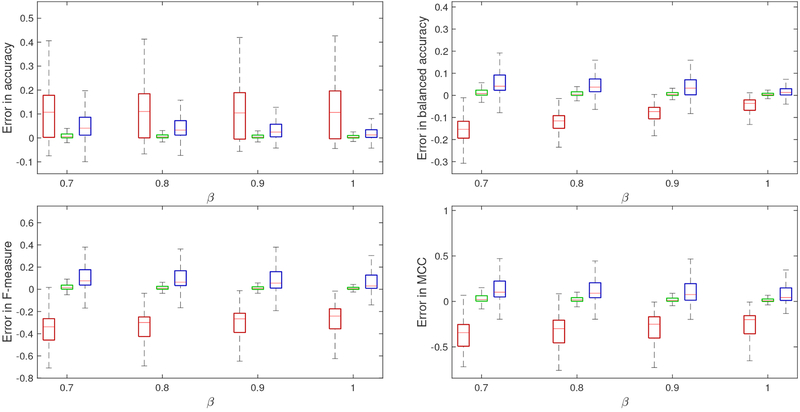
Error in the non-traditionally evaluated performance measures before and after correction for 14 biomedical data sets. PU represents the estimates on the **P**ositive **U**nlabeled data without bias-correction. CR and CE represent the bias-**C**orrected estimates with the **R**eal and **E**stimated values of *α* and *β*. In each run, the optimal decision threshold was selected first, to maximize the performance, and then the resulting performance was compared with the true performance at that same threshold. (Upper left) Classification accuracy: Eq. (12) was used for correction. All estimates were clipped between 0 and 1; (Upper right) Balanced accuracy: Eq. (13) was used for correction. All estimates were clipped between 12 and 1; (Lower left) F-measure: Eq. (14) was used for correction. All estimates were clipped between 0 and 1; (Lower right) Matthews Correlation Coefficient: the formula from Theorem 2.1 was used for a direct correction from the mcc^pu^ estimate. All estimates were clipped between −1 and 1. The x-axis is the true value of *β*, according to which the box plots were grouped.

We measured the difference between non-traditional and corrected performance against the traditional performance in each run. The traditional performance was considered to be “true”; it could be estimated because the positive-unlabeled setting was simulated on data sets where both positives and negatives were available. The corrected performance was presented twice: first with known (*α, β*) that were used to construct positive-unlabeled data sets and, second, with (*α, β*) themselves estimated from the positive-unlabeled data. The experimental results, summarized in a single box plot over all 14 data sets and all 50 runs, are shown in Figure 2. Non-traditionally estimated (without correction) bacc_max_, *F*_max_ and mcc_max_ significantly underestimate the traditional performance, whereasd acc_max_ significantly overestimates it. The errors generally deteriorate with the increasing level of noise (1 − *β*).

The corrected estimates attained much smaller error. While using the true values of *α* and *β* provided a near perfect recovery of the traditional performance, the estimated values generally resulted in a slightly overestimated traditional performance. We note however that we did not perform any model selection and parameter optimization during class prior and noise level estimation and, therefore, one could expect to observe an improved recovery after these steps. Manual inspection of the likelihood curves outputted by AlphaMax would also be recommended to increase confidence in the recovered performance estimates.

## 4. Conclusions

Estimating the performance of machine learning models is one of the critical yet understudied research directions in the biomedical sciences. Incorrect evaluation might have severe negative effects upon the deployment of machine learning tools and the perception of their usefulness in the nearby future, including in genetic counseling, precision medicine, clinical decision support, etc.^3–6^ This work therefore investigated the quality of performance evaluation in binary classification when training data best fits the positive-unlabeled setting.^18^ However, the generality of our methods is provided by the equivalence between training from noisy positive vs. unlabeled data and the so-called corrupt binary classification model, where it is assumed that both positive and negative examples are given, but that each data set is corrupted by a (potentially) different amount of label noise.

To characterize performance evaluation problems, we built on the previous work in machine learning^22,29^ to evaluate the quality of four estimated measures: accuracy, balanced accuracy, F-measure, and Matthews correlation coefficient. We found that the balanced accuracy and Matthews correlation coefficient are well-behaved, meaning that they provide certain important guarantees to the practitioner even when applied in the positive-unlabeled setting. For example, the optimal decision threshold for maximizing the performance does not change when the evaluation is shifted from the non-traditional to the traditional setting; furthermore, the performance in the traditional setting is always better than non-traditionally estimated. On the other hand, classification accuracy and F-measure provide fewer guarantees and require sophisticated understanding when deployed in practice.

To mitigate the problems associated with any of the above-mentioned performance estimation strategies, we first showed that the true (traditional) classification performance can be recovered with the knowledge of (1) the class priors in the unlabeled data and (2) the proportion of noise in the labeled data. We then used the AlphaMax algorithm^21,28^ to estimate both of these quantities in a nonparametric fashion and showed that the performance estimation process is significantly improved. As a practical guideline, we suggest that the deployment of machine learning models should be accompanied with both non-traditional and recovered traditional performance estimates along with the estimated values of *α* and *β*.

## Figures and Tables

**Table 1:** (a) Confusion matrix of $\hat{y}(x)$ on a labeled data set. (b) Standard estimation of *γ*, *η*, *π* and *θ*.

$$\begin{array}{cc} \begin{array}{ccc} & \begin{array}{l} \text{Predicted}\\ \text{positive} \end{array} & \begin{array}{l} \text{Predicted}\\ \text{negative} \end{array}\\ \text{Positive} & \text{tp} & \text{fn}\\ \text{Negative} & \text{fp} & \text{tn} \end{array} & \begin{array}{cccccc} \hat{\gamma } & = & \frac{\text{tp}}{\text{tp}+\text{fn}} & \hat{\pi } & = & \frac{\text{tp}+\text{fn}}{\text{tp}+\text{fn+tn}+\text{fp}}\\ \hat{\eta } & = & \frac{\text{fp}}{\text{tn}+\text{fp}} & \hat{\theta } & = & \frac{\text{tp}+\text{fp}}{\text{tp}+\text{fn}+\text{tn}+\text{fp}} \end{array}\\ \left(\text{a}\right) & \left(\text{b}\right) \end{array}$$

## Footnotes

b Even with exhaustive experimentation and no human error, the “negative” findings are rarely published.

c For convenience, we use terms density and distribution interchangeably.

d We only consider the *F*_*1*_ score in the family of F-measures.
